# Whole-Genome Sequence Analysis Reveals the Enterovirus D68 Isolates during the United States 2014 Outbreak Mainly Belong to a Novel Clade

**DOI:** 10.1038/srep15223

**Published:** 2015-10-15

**Authors:** Weihua Huang, Guiqing Wang, Jian Zhuge, Sheila M. Nolan, Nevenka Dimitrova, John T. Fallon

**Affiliations:** 1 Department of Pathology, New York Medical College, Valhalla, New York, United States of America; 2 Department of Pathology and Clinical Laboratories, Westchester Medical Center, Valhalla, New York, United States of America; 3 Division of Pediatric Infectious Disease, New York Medical College, Valhalla, New York, United States of America; 4 Philips Research North America, Briarcliff Manor, New York, United States of America

## Abstract

In the late summer and the fall of 2014, the United States experienced an unprecedented outbreak of enterovirus D68 (EV-D68) infections. During the outbreak, we collected nasopharyngeal swab specimens from patients in the Lower Hudson Valley of New York. Here, we conduct a retrospective study on the genomic diversity of EV-D68 strains. We first employ a metagenomic shotgun sequencing protocol on a total of 93 clinical samples, including 21 negative controls, the results of which allow assembly of 20 EV-D68 genomes, six complete and 14 near-complete. We then investigate their genetic relationships, along with additional 20 EV-D68 strains having whole-genome sequences publicly available. Our comparative genomic analysis uncovers that the majority (26/29) of EV-D68 strains circulating in the 2014 outbreak differ significantly from prior ones, have a main feature of three variables, C1817T, C3277A, and A4020G, and belong to a new clade. C3277A causes amino acid substitution T860N in the protease 2A^*pro*^ cleavage site between VP1 and 2A, whereas A4020G causes S1108G in a 3C^*pro*^ cleavage site between 2B and 2C. The two functional mutations may alter the proteases’ cleavage efficiency, leading to increased rate of viral replication and transmission. These provide insights into the evolution of epidemic EV-D68 strains.

Enteroviruses are small, non-enveloped RNA viruses. The genus *Enterovirus* of the family *Picornaviridae* includes seven species that commonly cause human disease: enterovirus A, B, C, and D and rhinovirus A, B, and C. Enterovirus D68 (EV-D68) is one of four serotypes assigned to enterovirus D. EV-D68 was first identified in California in 1962 from four children with pneumonia and bronchiolitis^1^. However, reports of respiratory disease due to EV-D68 were rare until 2009 when an upsurge commenced worldwide^2^,^3^. In 2014, the USA experienced an unprecedented nationwide outbreak of EV-D68. From mid-August 2014 to January 15, 2015, the Centers for Disease Control and Prevention (CDC) or state public health laboratories confirmed a total of 1,153 people in 49 states and the District of Columbia with respiratory illness caused by EV-D68 (http://www.cdc.gov/non-polio-enterovirus/outbreaks/ev-d68-outbreaks.html). Almost all of the confirmed cases were among children, many of whom had asthma or a history of wheezing. The clinical presentation of EV-D68 infections in the outbreak ranged from mild upper respiratory illness to severe respiratory complications requiring hospitalization and, in rare instances, subsequent death. As the 2014 outbreak unfolded, a possible association was also perceived between EV-D68 infection, polio-like acute flaccid paralysis, and cranial neuropathy in children^4^,^5^.

An enterovirus genome typically consists of a positive-sense single-stranded RNA of approximately 7,500 bases with a single open reading frame (ORF) encoding a polyprotein. The polyprotein ORF is flanked by a long untranslated region at the 5′ end (5′UTR) and a short untranslated region at the 3′ end. The 5′UTR contains a variable spacer region between a highly conserved internal ribosome entry site (IRES) and the polyprotein ORF. The polyprotein is processed co- and post-translationally to yield individual mature viral peptides, including structural VP1-VP4 from P1 capsid precursor; 2A protease (2A^*pro*^), 2B and 2C (ATPase) from P2 enzymatic precursor; and 3A, 3B (VPg), 3C protease (3C ^*pro*^), and 3D (RNA polymerase) from P3 enzymatic precursor. Whereas 2A^*pro*^ is responsible for the primary cleavage of the capsid precursor P1 from the non-structural region P2-P3 (between VP1 and 2A), 3C^*pro*^ performs eight cleavages within the polyprotein. In addition to processing of the viral polyprotein, 2A^*pro*^ and 3C^*pro*^ are also involved in shutting off host cell activities that help the virus evade host defense mechanisms and promote its own replication^6^,^7^.

There were only seven whole-genome sequences and five additional near-complete genome sequences of the virus publicly available before the EV-D68 outbreak in 2014. During and after the outbreak, several new genome sequences of circulating EV-D68 strains were reported^8^,^9^. As of the end of February 2015, there are 17 whole-genome sequences and 33 additional near-complete genome sequences of the virus in the NCBI GenBank database as well as in the NIAID Virus Pathogen Resource (http://www.viprbrc.org), including 20 from the present study. These genome sequences provide a useful resource for tracking and clustering of EV-D68 strains. To date, the study on the evolution and genomic characteristics of the virus in association with the rapid spread and high severity of disease in the 2014 outbreak is very limited.

In mid-August 2014, hospitals in Missouri and Illinois noticed an increased number of children with severe respiratory illness and asthma^10^. A similar pattern was observed in healthcare facilities in the Lower Hudson Valley of New York in September 2014. During the period of the EV-D68 outbreak, we collected a large number of nasopharyngeal swab specimens. By using these samples, we previously evaluated an EV-D68-specific real-time reverse transcription PCR (rRT-PCR) assay developed by the CDC and detected 72 specimens positive for EV-D68 infection^11^. Since the next-generation sequencing (NGS) technology has been applied successfully for the rapid identification of pathogens in clinical and public health settings, we employed a metagenomic shotgun sequencing (MSS) protocol on these 72 EV-D68-positive samples along with 21 negative controls. We achieved six complete and 14 near-complete EV-D68 genome sequences from these 93 clinical samples. We further explored the genomic diversity of EV-D68 strains and examined whether there were specific genetic elements associated with the USA outbreak in 2014.

## Materials and Methods

### Samples

This retrospective study was conducted as a part of hospital outbreak investigation of EV-D68-associated respiratory illness in the Lower Hudson Valley of New York State. A significant increase of pediatric patients with severe respiratory illness was observed at the Maria Fareri Children’s Hospital of Westchester Medical Center (WMC) in September 2014. In a period from September 18 to October 31, 2014, we collected 260 nasopharyngeal swab specimens that were reported positive for human rhinovirus/enterovirus (HRV/EV) by the FilmArray Respiratory Panel assay (version 1.7, BioFire Inc., Salt Lake City, UT) in the routine diagnostic testing at the clinical virology laboratory of the WMC. These 260 specimens were later subjected to an EV-D68-specific rRT-PCR assay followed by Sanger sequencing, in which partial sequences in VP-1 and 5′UTR of the enterovirus genome were amplified^11^. We identified 72 (27.7%) positive for EV-D68. The RNA samples from these 72 EV-D68-positive specimens, along with 13 randomly selected negatives, were further evaluated in this study using the NGS technology. For additional negative controls, we retrieved from −80 °C freezer eight nasopharyngeal swab specimens that were collected from September to December 2013. A total of 93 RNA samples were used in the present study.

Respiratory virus sample collection for this study occurred during the evolving, nationwide EV-D68 outbreak. EV-D68 testing and sequencing occurred in collaboration with local and state Departments of Health as part of the outbreak investigation. Methods were carried out in accordance with the Department of Health and Human Services CFR 45 Part 46 Protection of Human Subjects. The New York Medical College Institutional Review Board approved the study and granted a waiver for informed consent from patients.

### Next-generation sequencing

RNA was extracted directly from nasopharyngeal swab supernatants using the QIAamp Viral RNA Mini Kit (Qiagen, Valencia, CA), initially with carrier RNA added in nine samples and later with a minor modification that no carrier RNA was added in the remaining samples. One-fifth of RNA (12 μl from a total of 60 μl) was used for the first strand complementary DNA (cDNA) synthesis with the QuantiTect Reverse Transcription Kit (Qiagen), which included a genomic DNA elimination reaction at 42 °C for two minutes, a reverse transcription at 42 °C for one hour using random primer mix, and a final inactivation at 95 °C for three minutes, as per the manufacturer’s instruction. Half of the first strand cDNA (10 μl) was subject to the second strand cDNA synthesis at 16 °C for one hour using a cDNA synthesis system, which included the second strand synthesis buffer (1×, final), DNA polymerase I (10 U), RNase H (0.35 U), *E. coli* DNA ligase (1.25 U), and dNTP mixture (375 μM) (Life Technologies, Grand Island, NY) for each reaction. After enzymes denatured at 85 °C for 10 minutes, concentration of resulting double-stranded DNA (dsDNA) was measured using the Qubit dsDNA HS Assay Kit and Qubuit 2.0 Fluorometer (Life Technologies). Dual-indexed sequencing library was prepared using the Nextera XT DNA Sample Prep Kit (Illumina, San Diego, CA) with one nanogram of resulting cDNA and a long fragment protocol as per the manufacturer’s instruction. After normalization and pooling together, the final library of 93 samples was quantified with real-time PCR before loading into the MiSeq sequencing system (Illumina). Paired-end sequencing, 2 × 250 base pairs (bp), was performed using the MiSeq Reagent V2 (500 cycles) Kit (Illumina).

### Data Analysis

Contiguous sequences (contigs) were assembled *de novo* using the Velvet algorithm with optimized *k*-mers (version 1.0.9)^12^. The first and the most complete genome assembled, NY328, was used as a reference for further on-instrument alignment/mapping with the re-sequencing workflow of MiSeq Reporter (version 2.5, Illumina), in which BWA algorithm^13^ was applied and PCR duplicates were flagged. The large contigs (≥500 bp) were then manually evaluated, improved, and validated under the visualization of the Integrative Genomics Viewer^14^. Any hairpin loops at either 5′ or 3′ end of the assembled genome, likely formed in the second strand cDNA synthesis step, were removed. The resulting genome sequences, along with those EV-D68 genomes publicly available in GenBank, were compared using the Clustal X (version 2.1)^15^. The biostar94573 algorithm of jvarkit (https://github.com/linden/jvarkit/wiki/biostar94573) was used to generate a variant call format file (.vcf) from the CLUSTAL multi-sequence alignment file (.aln). The vcftools suite^16^ was used to generate genotypes for each individual strain from the .vcf file. The RStudio (www.rstudio.com) platform was employed, with the package adegenet (version 1.4–2)^17^ loaded. Principal Component Analysis (PCA) was employed to quantitatively assess the genetic diversity between EV-D68 strains using the squared Euclidean distances (function *dist*). Hierarchical clustering with complete linkage (function *hclust*) was used to define tight clusters. Discriminant Analysis of Principal Components (DAPC)^18^ was employed to identify genetic variables significantly associated with the 2014 EV-D68 outbreak in the USA. Both Pearson’s chi-square test and Fisher’s exact test were employed to estimate the *p*-value significance of genetic association for each variable identified.

## Results

### Twenty genome sequences of EV-D68 are obtained from a collection of nasopharyngeal swab specimens during the 2014 outbreak

To detect EV-D68 strains and to assess their genomic diversity during the 2014 outbreak of EV-D68 in the USA, we employed a MSS protocol directly on RNA samples isolated from nasopharyngeal swab supernatants, without any additional enrichment or amplification. With dual index coding for each sample, a total of 93 samples were pooled together on a single flow-cell for the MiSeq sequencing, generally yielding an average of 103,531 clusters passing filter per sample. Of these 93 samples, 72 were EV-D68-positive and 21 were negatives, as we detected previously using the rRT-PCR assay^11^. We used these negative controls to monitor if there was any cross-sample contamination. We identified a very low number of short reads aligned to the EV-D68 genome (≤3) in each EV-D68-negative sample, except 11 short reads (8/3) in sample NY266. Nevertheless, to date it is difficult for us to justify whether these short reads were from cross-sample contamination or not. The detection of EV-D68 in these 93 samples using our MSS protocol is summarized in Suppl. Table 1. Due to the uniqueness of each clinical sample (carrier RNA added or not, more RNA virus infection or less, more inhabiting microbiome or less, etc.), the number of short reads for each sample varied significantly, from hundreds to hundreds of thousands.

We first did *de novo* assembly for all these 93 samples using the Velvet algorithm. The sequencing data allowed assembly of six complete and 14 near-complete EV-D68 genomes from 20 patients. We deposited these resulting genome sequences in GenBank under accession no. KP745751-KP745770, BioProject PRJNA274680. The clinical characteristics of these 20 EV-D68 strains and their estimated sequencing coverage are demonstrated in Suppl. Table 2. We noticed that in our previous rRT-PCR assay these 20 samples had relatively low cycle threshold value (average Ct = 21.83) in comparison with the remaining 52 positives (average Ct = 28.36), indicating that these samples contained relatively higher concentration of EV-D68 particles.

Of the 20 assembled genomes, NY328 was the first one having a large assembled contig with the most complete genome of EV-D68. We then employed it as a reference for sequence alignment/mapping and EV-D68 detection (Suppl. Table 1), as well as annotation hereafter.

### Whole-genome sequence comparison demonstrates EV-D68 strains circulating in the 2014 USA outbreak belong to a novel clade

**Figure 1:**
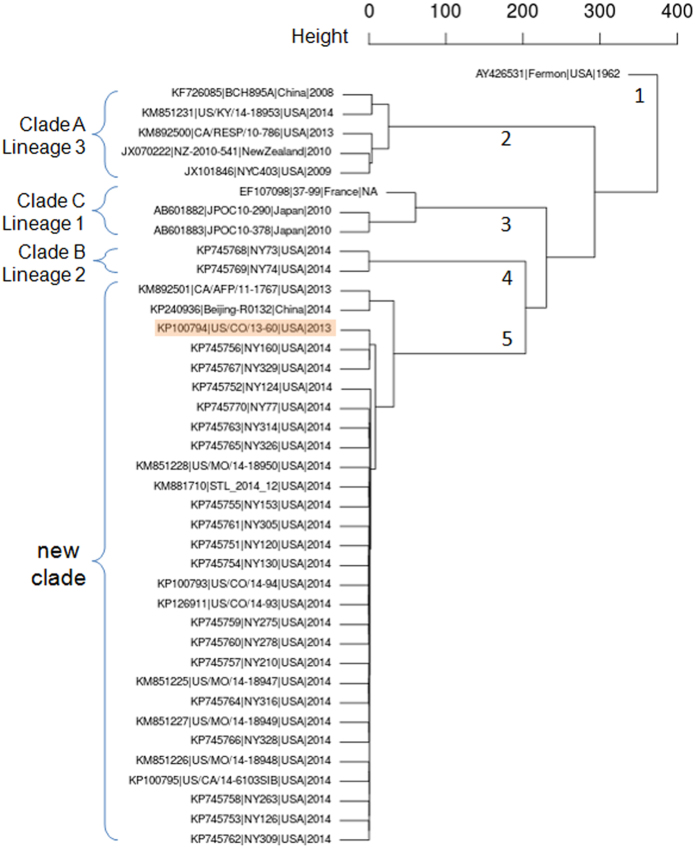
Hierarchical clustering with complete linkage of 40 strains based on the first four principal components. Clades or Lineages were defined previously on the basis of a partial VP1 sequence and a 5′UTR region sequence^2^,^3^. The five main clusters are shown in numbers, and Cluster 5 is indicated as a new clade on the basis of our whole-genome sequence analysis.

**Figure 2:**
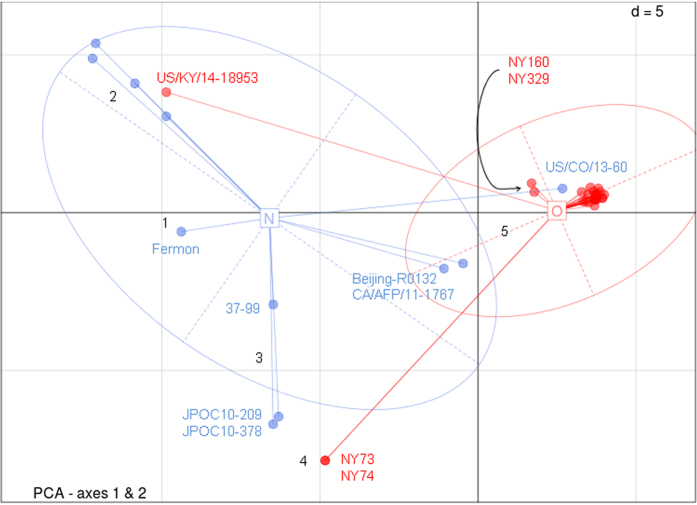
Scatter plots of 40 strains based on the first two principal components. Two groups are shown by colors and ellipses, while dots represent 40 individual strains. Red: EV-D68 strains in the USA in 2014—Group O; blue: EV-D68 strains before 2014 or outside of the USA—Group N. The five main clusters are shown in numbers.

To investigate the genomic diversity of EV-D68 strains, we employed PCA on a collection of 40 strains, 20 from the present study and another 20 from GenBank, each having a complete or near-complete genome sequence with a length of more than 7,200 bases (Table 1). Of these 40 EV-D68 strains, 29 were collected during the 2014 outbreak in the USA, whereas 11 were collected either outside the USA or before 2014. Accordingly, we defined them into two groups, Group O and Group N, respectively (Table 1). Through a multiple sequence alignment of these 40 strains, we identified a total of 1552 variables, among which we retained four principal components to capture more than 90% of the total variation. Based on these four principal components, we clustered the 40 EV-D68 strains hierarchically with complete linkage (Fig. 1). Demonstrated in Fig. 2 are the genetic relationships between these 40 strains in the two groups, on the basis of the first two principal components. In addition, we compared our clusters directly with three clades (or three lineages) that were defined previously based on phylogenetic analyses of a partial VP1 sequence and a partial 5′UTR region sequence^2^,^3^.

Of the 40 strains, we mainly identified five clusters: 1) strain Fermon, the first EV-D68 identified in 1962^1^ rooting the phylogeny tree in Fig. 1, has a complete spacer sequence (Suppl. Fig. 1); 2) five strains including a 2014 USA strain US/KY/14-18953, belonging to Clade A or Lineage 3 and shown in top-left section of Fig. 2, share a unique feature of three-nucleotide deletion in the VP1 region (Suppl. Fig. 2) and all have a deletion of 23-24 nucleotides in the spacer region (Suppl. Fig. 1); 3) a France strain 37–99 and two 2010 Japan strains (JPOC10-290 and JPOC10-378), belonging to Clade C or Lineage 1, have a 23-nucleotide deletion and a 12-nuleotide deletion in the spacer region (Suppl. Fig. 1); 4) strains NY73 and NY74, belonging to Clade B or Lineage 2^19^, are clustered closer to Cluster 5 as shown in Fig. 1 with four principal components but seem closer to Cluster 3 as shown in Fig. 2 with two principal components; and 5) the remaining 29 strains in the red ellipse in Fig. 2 differ significantly from those in the other clades and thereby should belong to a potential new clade (Fig. 1). Notably, the majority (26/29) of strains in Cluster 5 were from the EV-D68 outbreak in 2014 USA (Figs 1 and 2), despite being collected from a variety of geographic areas. Of most importance, Pearson’s chi-square test of these 40 strains in the five clusters indicates that the separation of Group O and Group N is significantly correlated to the main genomic features of these 40 strains (χ^2^ = 22.50, df = 4, *p* = 0.00016), suggesting there likely existed genetic elements associated with the EV-D68 outbreak in 2014 USA. The *p*-value of Fisher’s exact test for this correlation is 0.000030 (Suppl. Table 3).

### Genome-wide association study reveals three mutations are significantly associated with the EV-D68 outbreak in 2014 USA

**Figure 3:**
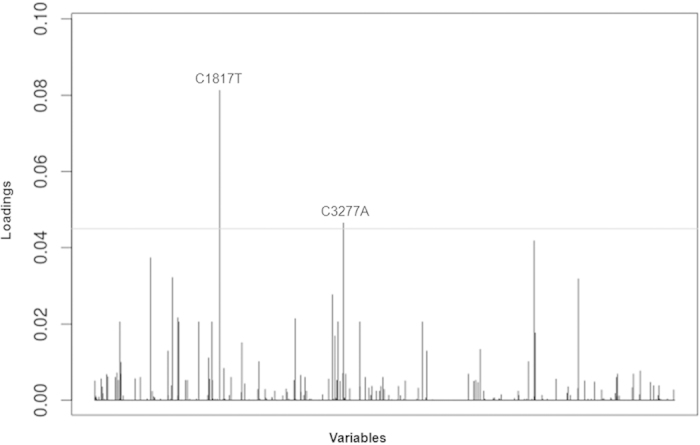
Contributions of variables to the discriminant analysis of principal components of the EV-D68 outbreak in 2014 USA. For the sake of clarity, only variables whose contribution is above an arbitrary threshold (0.045) are indicated and labeled by their positions aligned to reference genome NY328.

**Figure 4:**
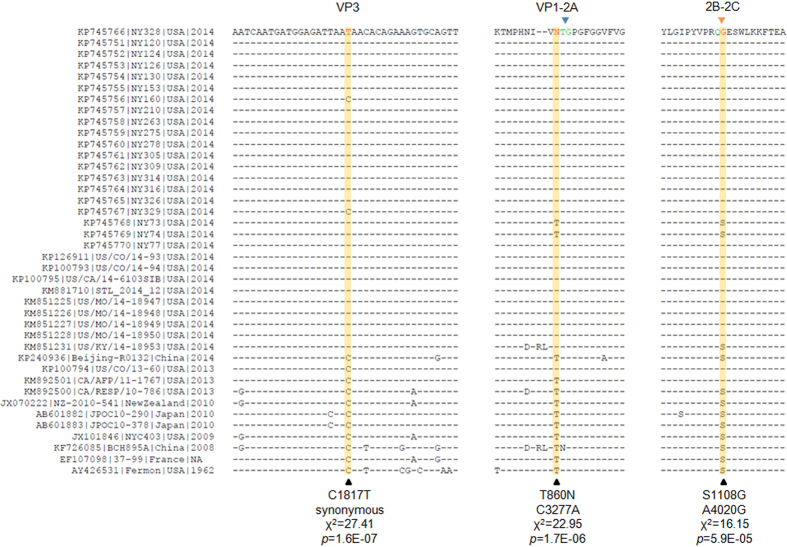
Multiple sequence alignments of 40 EV-D68 strains in reference to NY328. Left: alignment of nucleotide sequences adjacent to synonymous variable C1817T; middle: alignment of peptide sequences adjacent to T860N, caused by variable C3277A; right: alignment of peptide sequences adjacent to S1108G, caused by variable A4020G.

Next, we conducted a multivariate approach with DAPC to identify the genetic elements. We found two polymorphisms, C1817T and C3277A, had the largest contribution to the separation of these two groups, Group O and Group N (Fig. 3). The variables and positions were based on the multiple sequence alignment in reference to NY328 genome, the same hereafter. We found all strains in Group N contained 1817C, whereas all strains in Group O, except two (NY160 and NY329), contained 1817T (χ^2^ = 27.41, df = 1, *p* = 1.6E-07, Fig. 4 left panel). Polymorphism C1817T was synonymous and resided in the sequence region encoding VP3 peptide. In contrast, polymorphism C3277A caused an amino acid change from Thr to Asn (T860N) and resided in the sequence region encoding VP1 peptide. We found all strains in Group N except strain US/CO/13-60 contained 860Thr, whereas all strains in Group O except two (NY73 and NY74) contained 860Asn (χ^2^ = 22.95, df = 1, *p* = 1.7E-06, Fig. 4 middle panel). The *p*-values of Fisher’s exact test for the associations of these two variables are 3.4E-08 and 8.0E-07, respectively, as shown in Suppl. Table 4.

**Figure 5:**
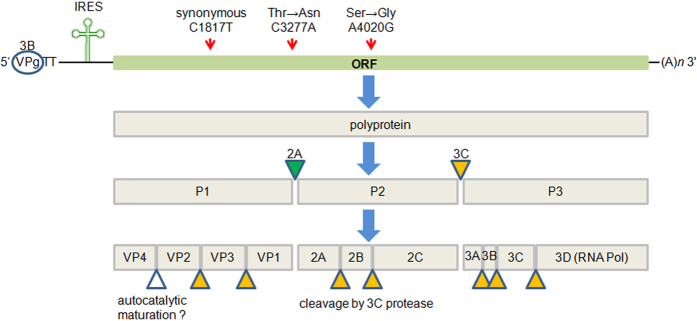
Genome structure of EV-D68 and maturation of 11 peptides from polyprotein. Yellow triangle: cleavage by 3C^*pro*^; green triangle: cleavage by 2A^*pro*^; white triangle: unknown autocatalytic maturation; red arrows: positions of the three identified variables on the genome.

**Figure 6:**
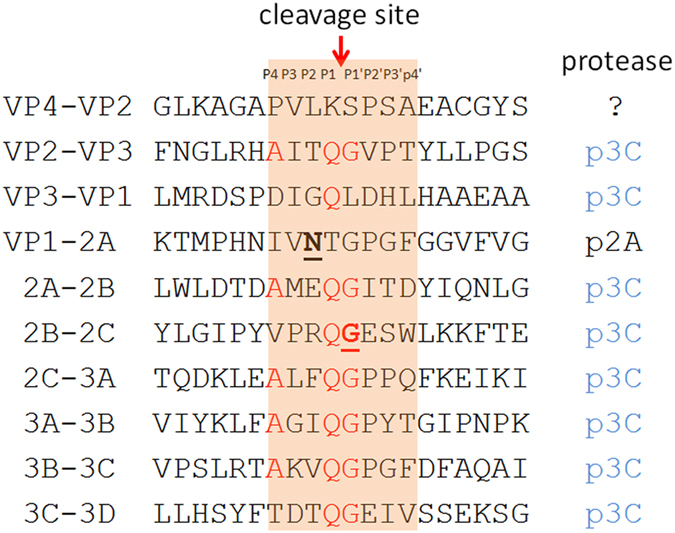
Alignment of NY328 peptide sequences adjacent to protease cleavage sites. Red: conserved motif AxxQ↓G for 3C^*pro*^ cleavage; bold and underlined: two identified variables (T860N and S1108G); red arrow: cleavage site of proteases.

The genome of EV-D68 encodes only one polyprotein, which matures into 11 peptides by cleavage with mainly two proteases, 2A^*pro*^ and 3C^*pro*^ (Fig. 5). The variable T860N was the second residue to the carboxyl-terminus of VP1 peptide, very proximal to the 2A^*pro*^ cleavage site between VP1 and 2A. To examine whether there was any additional variable at or proximal to the 3C^*pro*^ cleavage sites that would contribute significantly to the separation of our two defined groups, we aligned together the polyprotein sequences of these 40 strains and manually inspected all eight 3C^*pro*^ cleavage sites. We then identified variable S1108G, caused by non-synonymous polymorphism A4020G, residing at the amino-terminus of peptide 2C and right at the 3C^*pro*^ cleavage site between 2B and 2C. Only three of 29 strains in Group O contained 4020A, or 1108Ser; whereas two of 11 strains in Group N contained 4020G, or 1108Gly (χ^2^ = 16.15, df = 1, *p* = 5.9E-05, Fig. 4 right panel). The *p*-value of Fisher’s exact test for the association of this variable is 9.8E-06 (Suppl. Table 4). By aligning together the protease cleavage sites in NY328, we additionally identified a common motif AxxQ↓G conserved for EV-D68 3C^*pro*^ (Fig. 6). All eight P1 positions were occupied by residue Gln, seven of eight P1′ positions were Gly, whereas five of eight P4 positions at the 3C^*pro*^ cleavage sites were Ala.

To confirm our above results, we further re-evaluated the significance of these three genetic associations by including more strains available in the NIAID Virus Pathogen Resource. We searched for additional EV-D68 strains that had the sequence information regarding any one of the three mutations, from either PCR-sequencing results or genomic sequences. We compared their corresponding sequences with those of the 40 strains already aligned. Results of this statistical re-analysis with enlarged sample sets, shown in Table 2 in comparison with Suppl. Table 4, further supported the significant associations we obtained above. Of interest, we identified nine EV-D68 strains from Italy 2014 containing 3277A or 860Asn (AIX97184-AIX97192), similar to those from USA 2014, suggesting that the 2014 circulating EV-D68 strains outside the USA may share the same genomic features as those in the USA. Although we grouped these nine strains into Group N as per our definition, our previous significant association of the variable C3277A (or T860N) sustained.

Since both T860N and S1108G are functional polymorphisms, we finally examined whether the combination of these two variables was correlated to our partition of the 40 EV-D68 strains. The result from our Pearson’s chi-square test also showed significant (χ^2^ = 26.96, df = 3, *p* = 6.0E-06), suggesting that the two functional mutations might have interaction and synergistic effect on the 2014 outbreak of EV-D68 in the USA. The *p*-value of Fisher’s exact test for this association is 1.4E-06 (Suppl. Table 5).

## Discussion

The 2014 USA outbreak is the largest and most widespread EV-D68 outbreak investigated to date. In this retrospective study, we applied NGS technology to detect EV-D68 circulating in the Lower Hudson Valley of New York during the outbreak and to explore its genomic diversity. We employed a simple MSS protocol, which required only a small amount of starting specimen and no prior enrichment or amplification. Although the detection sensitivity of our MSS assay was relatively low, compared to the rRT-PCR method we employed before, we were able to detect EV-D68 in most of our positive samples using a simple on-instrument BWA alignment/mapping algorithm (Suppl. Table 1). With the rich sequence information obtained from NGS, we may process our MSS data with additional software, such as Kraken^20^,^21^ and SURPI^22^, in future for the identification of EV-D68 as well as other pathogenic viruses and/or inhabiting microbiome existing in all of our 93 clinical samples.

From the NGS data obtained, we assembled 20 complete or near-complete genomes of EV-D68 strains, which almost doubled the number of EV-D68 genomes publicly available. For a whole-genome comparison and a follow-up genetic association study, we included additional 20 EV-D68 strains from GenBank, each having a genome sequence of more than 7,200 bases. Alignment of these 40 whole-genome sequences followed by PCA with hierarchical clustering revealed that our collected EV-D68 strains were mainly clustered together with other EV-D68 strains circulating in the USA during the 2014 outbreak and the majority of them (26/29) belonged to a novel cluster, distinct from the clades or lineages defined previously (Figs 1 and 2). Only three exceptional strains were observed: US/KY/14-18953 from Kentucky belonged to Clade A or Lineage 3 (Fig. 1), although contained mutations C1817T and T860N, not S1108G (Fig. 4); NY73 and NY74 from New York belonged to Clade B or Lineage 2^19^, and contained mutation C1817T, but neither T860N nor S1108G (Fig. 4). Notably, the two outliers of our 20 strains, NY73 and NY74, are almost identical, with only one nucleotide difference in their genome sequences. The children infected by NY73 and NY74, respectively, were from the same city, geographically separated from the remaining 18 EV-D68 strains (Suppl. Table 2).

It is worthy to mention that previous clustering of either three clades (A, B and C) using the maximum likelihood algorithm^2^ or three lineages (1, 2 and 3) using the neighbor-joining algorithm^3^ was mainly based on the alignment of a partial VP1 sequence and a partial 5′UTR region sequence of EV-D68 genome. Although these clustering outcomes are comparable, we believe that results we present here from a comparison of sequences having full coverage on the whole genome are more precise, comprehensive and informative. We have noticed that Cluster 5 could be refined further to distinguish two strains (CA/AFP/11-1767 and Beijing-R0132) from the others (Figs 1 and 2). Additionally, a 2013 USA strain US/CO/13-60 in Group N is clustered together with strains NY160 and NY329 and is the only one shown in the top-right section of Fig. 2, together with the majority (26/29) of EV-D68 strains circulating in the 2014 USA. Based on our combined phylogenetic and genetic analyses, we speculate that the origin of the EV-D68 outbreak in 2014 USA is very likely related to the 2013 strain US/CO/13-60, which contains both T860N and S1108G mutations.

For the genetic association study on the 2014 outbreak of EV-D68 in the USA, we separated 40 EV-D68 strains into two defined groups, Group O and Group N, based on their collection time and places. Through a multivariate approach, we further identified three variables significantly contributing to the partition of these 40 strains (Fig. 4). Two of the three variables were non-synonymous, leading to amino acid changes at protease cleavage sites, T860N at position P2 of the 2A^*pro*^ cleavage site between VP1 and 2A and S1108G at position P1′ of the 3C^*pro*^ cleavage site between 2B and 2C. Previous work^6^ on the specificity of picornaviral proteases unveiled that glycine was uniquely required at the P1′ position of the 2A^*pro*^ cleavage site, whereas 3C^*pro*^ preferentially cleaved between Glutamine-Glycine (Q–G) pairs. At the 2A^*pro*^ cleavage site, threonine at position P2 was the most conserved residue after P1′-Glycine^6^. Therefore, it is very likely that the T860N replacement at position P2 would alter the efficiency of 2A^*pro*^ cleavage, which co-translationally releases the capsid precursor P1 peptide. Of important note, different enteroviruses may be quite selective about their substrate preferences and rates of cleavage. Recently, it was reported that EV-D68 3C^*pro*^ displayed different activities toward cleavage sites, very low toward VP3-VP1 with Glutamine-Leucine (Q–L) pair and 2B-2C with Glutamine-Serine (Q–S) pair, but high toward the others with Q–G pair^23^. Since 3C^*pro*^ has a cleavage preference over Q–G pairs^6^,^23^, the S1108G replacement, changing Q–S pair to Q–G pair, might increase the rate of 3C^*pro*^ cleavage between 2B and 2C, thereby altering the stability of peptide 2BC and the maturation of peptides 2B and 2C. Peptide 2B is implicated in altering the host cell membrane permeability and peptides 2B, 2C, and their common precursor 2BC are implicated in the production of the membranous vesicular structures on which viral replication takes place^7^. Further, a recent research demonstrated that enteroviral particles are packaged within phosphatidylserine vesicles, non-lytically releasing from cells and providing greater infection efficiency than free single viral particles^24^. Therefore, it is very possible that mutation S1108G may affect EV-D68 viral replication as well as transmission.

Our genetic analysis demonstrated variable T860N had a significant association with the occurrence of large EV-D68 outbreak in 2014 USA and variable S1108G had a relatively weak association. We speculate that these two variables are the main feature in characterization of EV-D68 strains circulating in the outbreak. Both variables are functionally related to protease cleavage activity and peptide maturation, hence they may have potential to impact viral replication and transmission. Moreover, combined together, the two functional variables may show synergistic effect, which could lead to EV-D68 dissemination and causing more severe disease. Nevertheless, it is noteworthy that the sample size for our population-based case-control genetic analysis is small, because resources for the whole-genome sequences of EV-D68 are limited. Therefore, caution should be taken in interpreting the results of our genetic association study. Further observation and experimental validation are required. Most recently, Greninger *et al.* reported six coding polymorphisms identified in new appearing EV-D68 strains, which included both T860N and S1108G mutations^5^.

The global emergence of EV-D68 since 2009 and the rapid evolution of the virus might have led to the large outbreak in 2014 USA. Spread of EV-D68 as a pathogen causing acute respiratory infection was also observed in other countries in 2014^19^,^25^,^26^,^27^,^28^. To date, the epidemiology and pathogenesis of EV-D68 infection remain elusive. Our present retrospective study represents one effort toward understanding of EV-D68 infection and the recent outbreak. With more detailed clinical features of patients available, a study on the correlation between the high severity of disease and the genomic elements may provide further insights. We believe in the near future the whole-genome sequence data will predict the outcome of EV-D68 infection and help clinicians in patient treatment.

## Additional Information

**How to cite this article**: Huang, W. *et al.* Whole-Genome Sequence Analysis Reveals the Enterovirus D68 Isolates during the United States 2014 Outbreak Mainly Belong to a Novel Clade. *Sci. Rep.* **5**, 15223; doi: 10.1038/srep15223 (2015).

## Supplementary Material

Supplementary Information

## Figures and Tables

**Table 1:** Information for 40 EV-D68 strains with complete or near-complete genomes publicly available, 20 of which are from this study.

Strain Name	GenBank Accession	Collection Date	Country	Sequence Length	Genome Completeness	Defined Group
NY120	KP745751	09/28/2014	USA	7332	complete	O
NY124	KP745752	09/29/2014	USA	7288	near	O
NY126	KP745753	09/28/2014	USA	7283	near	O
NY130	KP745754	09/30/2014	USA	7308	near	O
NY153	KP745755	10/02/2014	USA	7332	complete	O
NY160	KP745756	10/05/2014	USA	7307	near	O
NY210	KP745757	10/14/2014	USA	7332	complete	O
NY263	KP745758	09/18/2014	USA	7255	near	O
NY275	KP745759	09/19/2014	USA	7325	near	O
NY278	KP745760	09/19/2014	USA	7332	complete	O
NY305	KP745761	09/22/2014	USA	7325	near	O
NY309	KP745762	09/23/2014	USA	7268	near	O
NY314	KP745763	09/24/2014	USA	7308	near	O
NY316	KP745764	09/24/2014	USA	7333	complete	O
NY326	KP745765	09/25/2014	USA	7296	near	O
NY328	KP745766	09/26/2014	USA	7333	complete	O
NY329	KP745767	09/26/2014	USA	7321	near	O
NY73	KP745768	09/25/2014	USA	7289	near	O
NY74	KP745769	09/25/2014	USA	7219	near	O
NY77	KP745770	09/28/2014	USA	7308	near	O
Beijing-R0132	KP240936	2014	China	7332	complete	N
STL_2014_12	KM881710	2014	USA	7296	near	O
US/CA/14-6103SIB	KP100795	10/2014	USA	7287	near	O
US/MO/14-18947	KM851225	08/2014	USA	7331	complete	O
US/MO/14-18948	KM851226	08/2014	USA	7215	near	O
US/MO/14-18949	KM851227	08/2014	USA	7311	near	O
US/MO/14-18950	KM851228	08/2014	USA	7292	near	O
US/KY/14-18953	KM851231	08/2014	USA	7348	complete	O
US/CO/14-93	KP126911	09/2014	USA	7265	near	O
US/CO/14-94	KP100793	09/2014	USA	7265	near	O
US/CO/13-60	KP100794	11/2013	USA	7315	near	N
CA/RESP/10-786	KM892500	2013	USA	7341	complete	N
CA/AFP/11-1767	KM892501	2013	USA	7343	complete	N
NZ-2010-541	JX070222	06/23/2010	NZ ^*^	7320	near	N
JPOC10-290	AB601882	2010	Japan	7332	complete	N
JPOC10-378	AB601883	2010	Japan	7331	complete	N
NYC403	JX101846	09/2009	USA	7341	complete	N
BCH895A	KF726085	10/06/2008	China	7348	complete	N
37-99	EF107098	1999	France	7333	complete	N
Fermon	AY426531	1962	USA	7367	complete	N

NZ^*^: New Zealand.

**Table 2:** Statistics for associations of the three identified variables with the EV-D68 outbreak in 2014 USA, using an enlarged dataset from the NIAID Virus Pathogen Resource and both Pearson’s chi-square test and Fisher’s exact test.

Position (NY328)	Variant	Amino Acid	Group N	Group O	Total Strains	χ^2^	*p*-value	Fisher *p*-value
1817	C	Asn	23	4	62	40.14	2.4E-10	6.4E-12
	T	Asn	1	34				
3277	C	Thr	37	2	78	33.85	5.9E-09	7.4E-10
	A	Asn	11*	28				
4020	A	Ser	11	7	51	13.32	2.6E-04	1.4E-04
	G	Gly	3	30				

^*^9 of 11 are from Italy 2014.
