# Supplementary material for: Whole-Genome Sequence Analysis Reveals the Enterovirus D68 Isolates during the United States 2014 Outbreak Mainly Belong to a Novel Clade
Source: Sci Rep. 2015 Oct 15;5:15223. doi: 10.1038/srep15223 (PMC4606740; doi:10.1038/srep15223)
Supplement: Supplementary Information [file srep15223-s1.pdf]

Whole-Genome Sequence Analysis Reveals the Enterovirus D68 Isolates during the United States 2014 Outbreak Mainly Belong to a Novel Clade

Weihua Huang[1*], Guiqing Wang[1, 2], Jian Zhuge[2], Sheila M. Nolan[3], Nevenka Dimitrova[4], John T. Fallon[1, 2]

[1] Department of Pathology, New York Medical College, Valhalla, New York, United States of America

[2] Department of Pathology and Clinical Laboratories, Westchester Medical Center, Valhalla, New York, United States of America

[3] Division of Pediatric Infectious Disease, New York Medical College, Valhalla, New York, United States of America

[4] Philips Research North America, Briarcliff Manor, New York, United States of America

* Corresponding author

Email: weihua_huang@nymc.edu

Supplemental Materials:

Suppl. Fig. 1 Multiple sequence alignment of 40 strains in the 5'UTR spacer region. In reference to the genome sequence of strain Fermon, two main deletions are identified, 23-24 nucleotides and 12-13 nucleotides, respectively. The five main clusters are shown in numbers. Red arrow and highlighted sequence: translation start codon; *: consensus nucleotide.

Suppl. Fig. 2 Multiple sequence alignment of 40 strains in a VP1 region. Only the five strains in Clade A or Lineage 3 have the three-nucleotide deletion, i.e. one amino acid deletion. The five main clusters are shown in numbers. *: consensus nucleotide.

Suppl. Table 1. Summary of EV-D68 detection in a total of 93 samples using our metagenomics shortgun sequencing protocol, in comparison with that using the rRT-PCR assay previously. Cluster PF: clusters passing the filter; Cluster/Align: paired reads (read1/read2) aligned to the reference genome NY328; "-": negative controls with EV-D68 undetectable under rRT-PCR.

Sample	Cluster PF	Cluster/Align	RT-PCR	Sample	Cluster PF	Cluster/Align	RT-PCR
NYA1[c]	51	0/0	19.88	NY263	177739	899/859	21.00
NY56[c]	36601	6/5	27.55	NY265	155960	115/111	25.07
NY58[c]	5231	7/6	29.47	NY266	52211	8/3	-
NY67[c]	40256	2/1	31.85	NY268	45284	2/2	27.62
NY68[c]	7013	0/0	28.59	NY269	60968	0/0	-
NY69[c]	26250	2/1	32.22	NY273	102258	7/6	32.02
NY70[c]	17345	2/3	28.04	NY274	139884	27/26	29.13
NY72[c]	20027	92/94	21.32	NY275	60115	1379/1294	23.49
NY73[c]	7800	415/388	18.71	NY278	27890	2398/2298	23.04
NY74	41716	729/628	20.96	NY281	37310	0/0	-
NY77	44910	590/563	19.85	NY284	2452	0/0	-
NY104	62748	3/3	30.94	NY287	36643	2/1	33.86
NY105	13077	0/0	33.41	NY288	121863	1/1	33.68
NY107	49126	150/134	24.52	NY289	50456	289/280	23.11
NY109	3454	0/0	35.10	NY291	70759	26/25	29.90
NY110	44289	1/0	31.96	NY293	96547	30/28	26.65
NY120	113245	1760/1755	20.09	NY295	50532	43/40	24.21
NY121	6758	0/0	-	NY300	136866	57/48	27.36
NY122	11429	0/0	-	NY301	258728	7/4	30.94
NY123	65604	0/0	32.21*	NY302	263861	319/309	24.97
NY124	191261	662/611	23.30	NY303	223897	4837/4537	19.88
NY126	196033	523/491	22.99	NY305	138637	867/810	24.65
NY129	214899	0/1	33.63	NY306	95802	5/3	32.08
NY130	105283	746/759	22.17	NY309	166518	1931/1855	22.68
NY132	158789	14/13	28.46	NY314	178162	647/609	23.12
NY134	148534	8000/7468	24.03	NY315	80372	0/1	-
NY135	217149	0/0	35.30	NY316	164816	1530/1473	21.96
NY136	155035	6572/6266	18.11	NY318	54265	37/35	27.21
NY139	139267	66/63	24.66	NY319	41091	325/318	26.25
NY153	131194	1520/1473	20.19	NY321	228798	7071/6707	17.94
NY156	11410	1/1	34.33	NY324	46442	2/0	35.09
NY160	71142	387/366	21.25	NY326	47022	2903/2793	21.80
NY165	230482	18/12	26.06	NY328	175316	850/812	23.73

NY171	474420	6/4	30.89	NY329	240676	3537/3342	20.24
NY177	295373	1/0	-	NY331	114788	25/26	29.10
NY178	82310	769/735	24.16	NY333	54681	4/4	31.27
NY179	128909	0/0	-	NY339	35206	1/0	-
NY181	354497	1/1	30.69	NY342	94906	0/0	-
NY193	178338	51470/48986	15.12	NY364[n]	22519	2/1	-
NY197	44174	78/76	26.86	NY365[n]	45136	0/0	-
NY207	109842	2/1	-	NY366[n]	30495	1/1	-
NY209	11194	179/169	27.36	NY367[n]	55262	0/0	-
NY210	23244	4105/3924	21.41	NY368[n]	49998	0/0	-
NY236	28102	0/1	33.72	NY369[n]	43191	0/0	-
NY241	156380	48/45	30.31	NY370[n]	261433	1/0	-
NY247	217170	1/0	39.76	NY371[n]	108221	1/0	-
NY262	191094	1/1	29.26				

c: carrier RNA used in RNA elution; n: 2013 sample; *: false positive confirmed by Sanger sequencing

Suppl. Table 2. Clinical characteristics of 20 EV-D68 strains with complete or near-complete genome sequenced and their estimated genome coverage.

Sample ID	Patient			Estimated Genome Coverage
	Age	Sex	City	
NY120	2	M	Yonkers	93
NY124	0.8	M	Mohegan Lake	31
NY126	10	M	Goshen	22
NY130	2	F	Yonkers	43
NY153	4	F	Yonkers	73
NY160	11	F	Garrison	17
NY210	5	M	Poughkeepsie	185
NY263	11	F	Mount Vernon	44
NY275	6	M	Middletown	51
NY278	12	M	Tappan	86
NY305	5	M	West Harrison	38
NY309	0.9	M	Briarcliff Manor	91
NY314	1	M	Jefferson Valley	27
NY316	3	F	Briarcliff Manor	71
NY326	9	M	Pelham	123
NY328	0.5	M	Newburgh	39
NY329	8	M	West Harrison	160
NY73	3	F	Ossining	10
NY74	4	F	Ossining	21
NY77	5	F	New Paltz	29

Suppl. Table 3. Statistics for correlation of main genomic features in 40 strains with the EV-D68 outbreak in 2014 USA, using both Pearson's chi-square test and Fisher's exact test.

	Cluster 1	Cluster 2	Cluster 3	Cluster 4	Cluster 5	Total	χ^2	*p*-value	Fisher *p*-value
**Group N**	1	4	3	0	3	11	22.50	1.6E-04	3.0E-05
**Group O**	0	1	0	2	26	29			

Suppl. Table 4. Statistics for associations of the three identified variables with the EV-D68 outbreak in 2014 USA, using both Pearson's chi-square test and Fisher's exact test.

Position (NY328)	Variant	Amino Acid	Group N	Group O	Total Strains	χ^2	*p*-value	Fisher *p*-value
1817	C	Asn	11	2	40	27.41	1.6E-07	3.4E-08
	T	Asn	0	27				
3277	C	Thr	10	2	40	22.95	1.7E-06	8.0E-07
	A	Asn	1	27				
4020	A	Ser	9	2	40	16.15	5.9E-05	9.8E-06
	G	Gly	2	27				

Suppl. Table 5. Statistics for association of the combined two functional variables C3277A and A4020G with the EV-D68 outbreak in 2014 USA, using both Pearson's chi-square test and Fisher's exact test.

	C3277-A4020	A3277-A4020	C3277-G4020	A3277-G4020	Total Strain	χ^2	*p*-value	Fisher *p*-value
**Group N**	9	0	1	1	11	26.96	6.0E-06	1.4E-06
**Group O**	2	1	0	26	29			
